# Feature space learning model

**DOI:** 10.1007/s12652-018-0805-4

**Published:** 2018-05-09

**Authors:** Renchu Guan, Xu Wang, Maurizio Marchese, Mary Qu Yang, Yanchun Liang, Chen Yang

**Affiliations:** 1 Key Laboratory for Symbol Computation and Knowledge Engineering of National Education Ministry, College of Computer Science and Technology, Jilin University, Changchun 130012, China; 2 Department of Engineering and Computer Science, University of Trento, 9I-38123 Povo, Italy; 3 MidSouth Bioinformatics Center and Joint Bioinformatics, University of Arkansas at Little Rock and University of Arkansas Medical Sciences, Little Rock, AR 72204, USA; 4 College of Earth Sciences, Jilin University, Changchun 130061, China; 5 Zhuhai Laboratory of Key Laboratory of Symbolic Computation and Knowledge Engineering of Ministry of Education, Zhuhai College of Jilin University, Zhuhai 519041, China

**Keywords:** Feature space learning, Semi-supervised learning, Affinity Propagation, k-means

## Abstract

With the massive volume and rapid increasing of data, feature space study is of great importance. To avoid the complex training processes in deep learning models which project original feature space into low-dimensional ones, we propose a novel feature space learning (FSL) model. The main contributions in our approach are: (1) FSL can not only select useful features but also adaptively update feature values and span new feature spaces; (2) four FSL algorithms are proposed with the feature space updating procedure; (3) FSL can provide a better data understanding and learn descriptive and compact feature spaces without the tough training for deep architectures. Experimental results on benchmark data sets demonstrate that FSL-based algorithms performed better than the classical unsupervised, semi-supervised learning and even incremental semi-supervised algorithms. In addition, we show a visualization of the learned feature space results. With the carefully designed learning strategy, FSL dynamically disentangles explanatory factors, depresses the noise accumulation and semantic shift, and constructs easy-to-understand feature spaces.

## 1 Introduction

In the era of big data, tasks such as natural language processing and ImageNet large scale visual recognition competition make that it is not enough if we just rely on simple parametric models, because they cannot capture enough complexity of interest unless provided with the appropriate feature space (Bengio et al. 2013). However, how to explore and generate the feature space to support effective machine learning is a major question. Recently, much of the actual effort in deploying deep learning algorithms such as deep belief networks (Jiang et al. 2016), auto-encoders (Hinton and Salakhutdinov 2006), convolutional neural network (Esteva et al. 2017) and recurrent neural networks (Zhang et al. 2018) goes into exploring feature space and learning good representations; however, most of the deep architectures are too challenging to train effectively. Another problem lies in disentangling and explaining the highly abstracted concepts or representations obtained from deep learning. The source of their performance is still lack of interpretability (Karpathy et al. 2015) (See Table 1).

Meanwhile, among the data mining techniques, clustering plays an important role in exploratory recommendation systems (Bobadilla et al. 2013), public opinion analyses (Semetko and Valkenburg 2000), and information retrieval areas (Frakes and Baeza-Yates 1992). Many clustering applications can be found in image segmentation, object recognition, video tracking, and etc. (Jain et al. 1999; Huang et al. 2018; Wu et al. 2018). Instead of only using the unlabeled sources, semi-supervised algorithms have attracted considerable attention because they can learn from a combination of labeled and unlabeled data for better performance (Wang et al. 2012; Guan et al. 2011). In semi-supervised clustering, the intuitive purpose is to use not only the available information from labeled data during clustering procedures, but also clues from unlabeled data to estimate data distribution. To exploit unlabeled data, most of the frameworks introduce the following two prior assumptions of consistency: (1) nearby points are likely to have the same label; and (2) points on the same structure (typically referred to a cluster or a manifold) are likely to have the same label (Xue et al. 2011). However, the biggest risk for directly using unlabeled samples is that the cluster could be adulterated with untrusted samples in its earlier stage. These bad pieces of information accompanied with good will propagate and be amplified in the following procedures and in other samples, which is similar to positive feedback in digital electronics and the Matthew effect in social psychology. Therefore, it is obvious that unlabeled points should be explored carefully because even the supervised classification performance can be degenerated by wrong unlabeled information (Li and Zhou 2015). In addition, most existing semi-supervised methods are lack of the ability for handling high-dimensional data (Tang et al. 2007).

To make good use of daily growing data (most of them are unlabeled samples) and avoid the wrong information propagation risk, we propose a novel feature space learning (FSL) model, which can perform adaptive feature space upgrading while fulfilling clustering. It is based on the hypothesis that unlabeled samples can provide useful information on distribution estimation (cluster center estimation) over feature space. Therefore, the new model creates label propagation in unlabeled texts with the help of clustering. Then, the incoming newly labeled samples are selected based on the objective function, which most of the clustering algorithms try to minimize. Moreover, a feature selection method inspired by a universal regularity for human language-Zipf’s law (Zipf 1949) and word burstiness (Kleinberg 2002) is developed to further control the risks. This model relies on the universal rules for human language which are named as Zipfs law and word burstiness. Because it uses an algorithm instead of functional mapping to dynamically delineate the feature spaces, FSL is quite different from the mean-shift algorithm, which is another feature space model in image segmentation and video tracking (Comaniciu and Meer 2002; Leichter 2012). This model combines prior information and an assumption of consistency, which could not only embed the labeled information in similarity measurements, but also guide the clustering procedures.

To illustrate the performance of our model, we applied FSL to two classical clustering algorithms and implemented four FSL algorithms: feature space seeded k-means (FSSK-means), feature space constrained k-means (FSCK-means), feature space affinity propagation (FSAP) and feature space seeds affinity propagation (FSSAP). Experiment was conducted with two benchmark data sets to demonstrate the effectiveness of the proposed algorithms. As a result, topical feature space for each cluster can be found accompanying the end of clustering. With the learned feature space, we can obtain a simple and compact representation of the data.

## 2 Feature space learning model

Studying the statistical properties and universal regularities of written texts can dig out clues about how our brains process information and model language computationally (Serrano et al. 2009). Among the studies in this area, the most notable regularities are Zipf’s law and the bursty nature of words.

Zipf’s law is one of the best-known universal regularities on word frequencies, wherein the frequency of terms *n_i_* in a collection decreases inversely to the rank *r* of the terms: *n_i_* ~ 1/*r_i_* or $P(n_{i})\sim n_{i}^{-\alpha }\alpha \approx 2$ which indicates *n_i_* is approximated by the power law. It applies to collections of texts in virtually all languages.

Due to the bursty nature of human behavior (Barabasi 2005) and the fact that bursty nature of rare words is connected with the topical organization of texts (Griffiths and Steyvers 2004), word bursts have attracted more and more attention. It is depicted as making a word *f* more likely to reappear in document *d* if it has already appeared, compared to its overall frequency across the collection (Serrano et al. 2009). Interestingly, those rare words are more evident with this property and they are more likely to be topical. To mine the potential information and use the consistency assumption, we introduced Zipf’s law and word bursts to control the potential risk while learning from both labeled and unlabeled samples.

Based on Zipf’s law and word bursts, the mainframe of the new feature space learning model is presented. Our hypothesis is that unlabeled samples can provide information about the distribution estimation (cluster center estimation) over feature space. For example, suppose that we determine that the word “computer” in a labeled document tends to be an important feature for its cluster vector. The most widely used text-mining model is the vector space model, which treats a document as a bag of words/phrases and uses plain language words as features. If we use this feature or “computer” to estimate the clusters of many unlabeled documents, we could find that the word “graphics” occurs frequently in the unlabeled examples that are now believed to belong to the “computer” cluster. In contrast, based on the prior assumption of consistency, it could also be expected that points (documents) with the same label are likely to share same or similar feature space. Therefore, the unlabeled samples can provide additional informative features to construct new feature space to provide further cluster estimation or change the cluster center vector to be more representative. However, based on the above hypotheses, there is a potential risk that noisy information may be picked out. To avoid the risk, we designed two constraint strategies–Zipfs’ law and word bursts in our feature space learning (FSL) model to optimize the objective function.

**Fig. 1:**
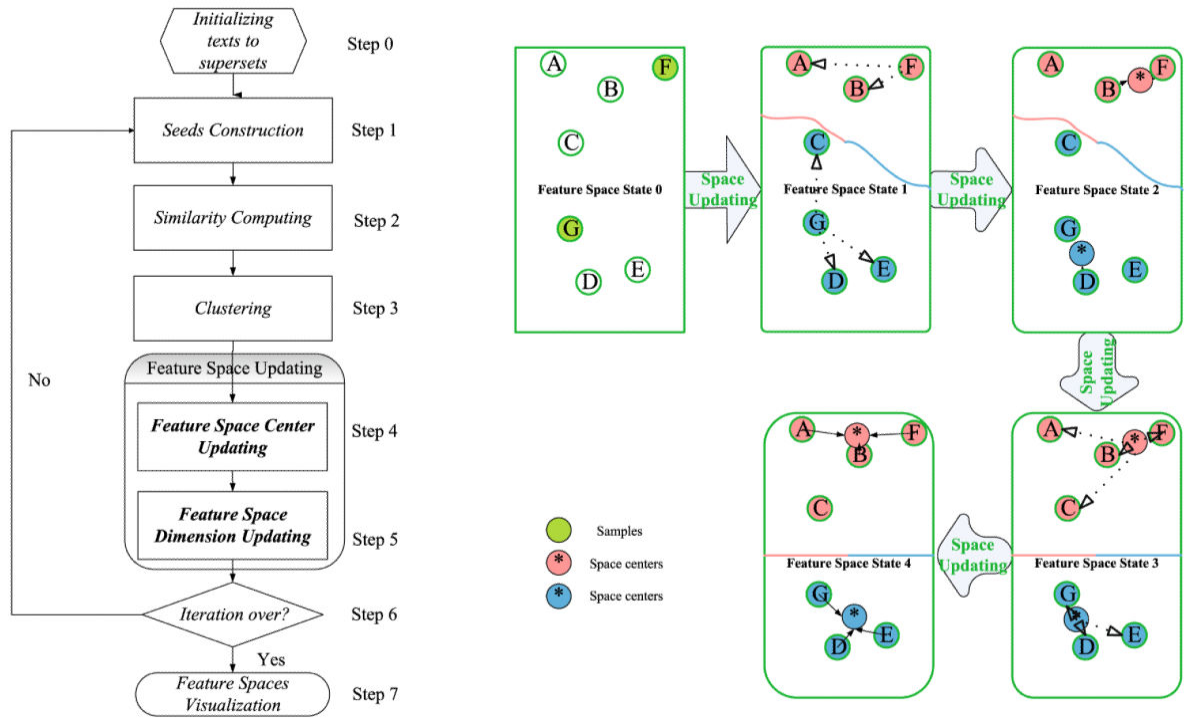
Flowchart of feature space learning model and feature space updating diagram

To clarify the FSL model and procedures, all the cluster processes are illustrated in Fig. 1. Here, all the detailed explanations are depicted as follows:

1. Initialization: Initialize data to supersets to convert all the texts into vectors. Let *D* {*d*_1_, *d*_2_, ⋯, *d_N_*} be a set of samples. Suppose that *d_i_* and *d_j_* are two objects in *D*; they can be represented as: $d_{i}=\{\langle f_{i}^{1},n_{i}^{1}\rangle ,\langle f_{i}^{2},n_{i}^{2}\rangle ,\cdots ,\langle f_{i}^{L_{i}},n_{i}^{L_{i}}\rangle \}$ $d_{j}=\{\langle f_{j}^{1},n_{j}^{1}\rangle ,\langle f_{j}^{2},n_{j}^{2}\rangle ,\cdots ,\langle f_{j}^{L_{j}},n_{j}^{L_{j}}\rangle \}$. where $f_{i}^{l}$ and $f_{j}^{m}(1\leq l\leq L_{i},1\leq m\leq L_{j})$ represent the *l*th feature of *d_i_* and the *m*th feature of *d_j_*, respectively. $n_{i}^{l}$ and $n_{j}^{m}$ are their feature values. *L_i_* and *L_j_* are the number of the objects’ features.
2. Seed construction: An intuitive way is to use the few-labeled samples. However, after step 4 and step 5, the seeds can be updated with rules.
3. Similarity computation: Different similarity metrics can be selected according to different data, such as cosine coefficient for texts and Euclidean distance for images.
4. Clustering: In this step, several classical clustering algorithms such as k-means and affinity propagation in our case can be adopted.
5. Feature Space control: It is the key procedure of our model and is designed to avoid wrong updating with noise data and features. The right part of Fig. 1 is a diagram of feature space updating (The change of frame and arrows indicate the feature space tranformation). It is described in detail in the next section.
6. The termination condition judgment. If clusters are not changed for several iterations or the maximum number of iterations value is reached, then the clusters and their topic feature space are generated.

When we face a real problem, three things should be emphasized in the feature space updating: first, how to update feature space centers.

**Definition 1** The sample d* is a trust sample for the *k*th cluster (*Trust_k_*), if

(1) $$d^{*}=\underset{d\in {\mathrm{Cluster}}_{k}}{\arg\;\max}Mem(d,k),\;1\leq k\leq K$$

where *K* is the number of clusters, *Mem*(*d*, *k*) is the membership function which indicates the extent of sample *d* belonging to cluster *k*. Different clustering algorithms try to maximize different membership functions, or to be equivalent, to minimize an object function opposed to membership functions. Therefore, trusted samples are selected by the algorithms and will provide useful update information for centers in the following processes. The count of “trust samples” could vary according to different applications (such as image processing, gene finding and so on) and requirements.

Moreover, the feature space dimension updating step provides another “firewall” for the security of adaptive information obtained from trust samples. It is believed that not all the features in the input space are important for clustering. This is because some of the features may be redundant or irrelevant to the cluster topics. Some may even misguide the clustering result, especially when the irrelevant features outnumber the relevant ones. Therefore, a great number of feature selection methods have been proposed, which could not only decrease the time and space complexity but also achieve improvements on clustering results. Instead of dimension reduction, we focus on the rich-information feature (e.g. words or key-phrases for text clustering) finding. We should emphasize that these features are selected from both labeled and unlabeled sources to generate a new space but not to reduce the dimension as an original purpose of feature selection for text mining. It is implemented to control the unlabeled utilization risk and select the burst items for providing adaptive information.

As the second issue, feature space updating needs an effective risk control strategy to avoid the risk of untrusted labels; meanwhile, the risk control strategy cannot be too complex to avoid the high computation complexity. Here we propose a simple but effective feature selection method with linear computational complexity based on Zipf’s law and word burstiness. It borrows the ideas from the bursty nature of words and contributes to further extension, which indicates that bursty words in each individual text (with stop words or function words removed) are potential to be the bursty words in the corresponding cluster. These words are more informative and contain more relationships with each other. Furthermore, they could be the major part of the topic feature space. We call these words as rich-information features (RIFs).

**Definition 2** Assume sample d* is a trust sample of the kth cluster, and the lth feature *f_l_* is a feature of sample d*. Then *f_l_* is a rich-information feature (RIF) for cluster k, if

(2) $$n_{*}^{l}\geq \eta \sum_{l=1}^{L_{*}}n^{l}∕L_{*},m_{k}^{i}\geq \mu \sum_{p=1}^{P_{k}}m_{k}^{p}∕P_{k}$$

where $n_{*}^{l}$ is the frequency of feature *f_i_* in trust sample d*, $m_{i}^{k}$ is the frequency of feature *f_i_* in the *k*th cluster, L_*_ and *P_k_* indicate the number of features in both d* and the *k*th cluster center while *η* and *μ* are two control parameters, respectively.

Definition 2 is a double constraints problem. It is derived from mutual information in information theory. The *RIF* selecting method is easily scaled to a “big” dataset, because of its linear computation complexity *O*(*M* + *L*).

Third, after the harsh feature selection, another rule is developed for updating weight in the adaptive processes to optimize the feature space. The feature space is constructed by adding rich-information features of trust samples into the original space iteratively. Therefore, the feature space can be considered as a linear combination of the vector of trust sample *RIF* and the original feature space. It should be noticed that the trust samples have different confidence levels at different iterations. Simply, the confidence of an *RIF* in *t* iteration is assigned as

(3) $${\mathrm{conf}}_{t}=(T-t)∕T$$

where *t* is the iteration times and *T* is the total number of iterations. Herein, the *RIF*s of the unlabeled samples’ contribution for cluster feature space is decreased linearly with iteration *t*. Denote the total confidence from the beginning until the *t*th iteration as

(4) $${\mathrm{conf}}_{T}=\sum_{i=1}^{t}{\mathrm{conf}}_{i}=\sum_{i=1}^{t}\frac{T-t}{T}=t\left(1-\frac{t+1}{2T}\right)$$

**Fig. 2:**
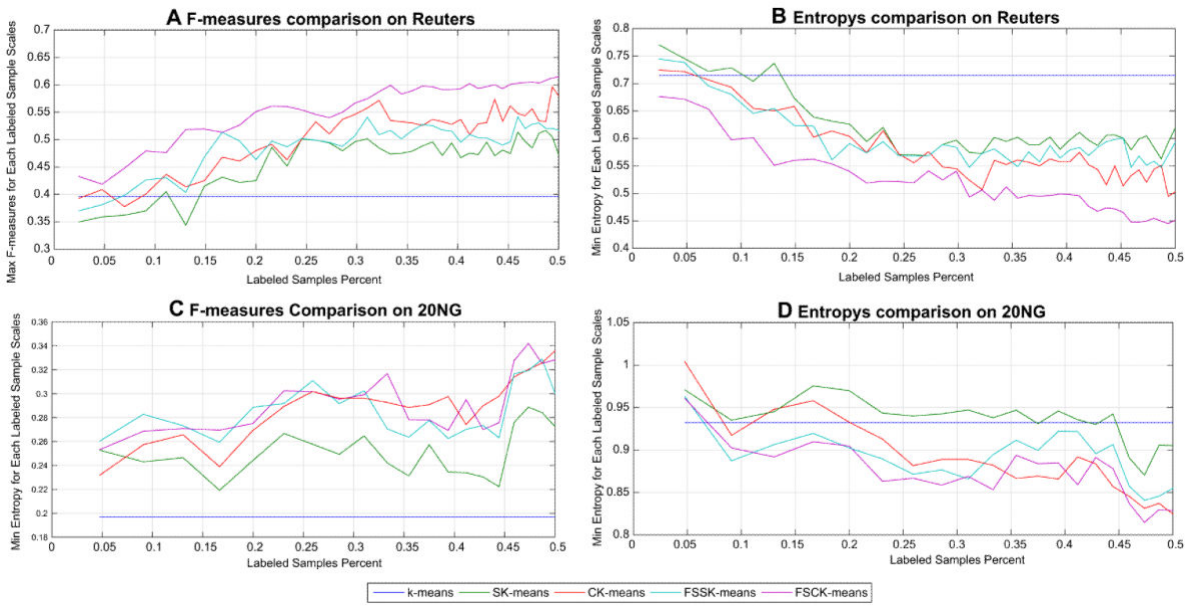
Comparison results of K-means based methods

Then, for an RIF *f*_i_ in trust sample d* of cluster *k* in the *t*th iteration, the weight updating rule (See step 5 in Figure 2) is set as follows:

(5) $${\left(w_{k}^{i}\right)}^{t}=\frac{conf_{t}}{conf_{T}}\times {\left(w_{k}^{i}\right)}^{t-1}+\frac{conf_{t}}{conf_{T}}\times n_{*}^{i}$$

where ${\left(w_{i}^{k}\right)}^{t}$ is the weight of *f*_i_ (a *RIF*) in cluster *k* for the *t*th iteration. In addition, considering the consistency assumption of semi-supervised learning, the similarity matrix needs to be updated for those new labeled samples. The matrix is re-computed as follows: If *i* and *j* belong to same cluster at the *t* iteration, their distance is set to the minimum; otherwise, the distance becomes the maximum. In particular, at the beginning of the algorithm, the similarities among all the labeled objects are also computed with this rule.

To examine the effectiveness of FSL, starting from two classical algorithms (k-means and affinity propagation) (Frey and Dueck 2007), we represent four FSL algorithms. These algorithms are named as feature space seed k-means (FSSK-means), feature space constrained k-means (FSK-means), feature space affinity propagation (FSAP), and feature-space-seeds affinity propagation (FSSAP).

### 2.1 K-means based FSL models

The main idea of k-means is to optimize the objective function:

(6) $$G(x)=\sum_{k=1}^{K}\sum_{d_{i}\in {\mathrm{Cluster}}_{k}}{||d_{i}-c_{k}||}^{2}$$

where *K* is the cluster number, *d_i_* is a sample involved in cluster *k* and *c_k_* is the cluster center, respectively. After the initialization with labeled samples and a complete k-means clustering, the next step is to select the trust samples. According to Eq. (6), the membership function in Eq. (1) should minimize the distance of the relative sample with its cluster center. Then the trust sample selection method could be represented by:

(7) $$d^{*}=\underset{d\in {\mathrm{Cluster}}_{k}}{\arg\;\min}{||d-c_{k}||}^{2},1\leq k\leq K$$

In the next step, those features satisfied in Eq. (2) are selected as RIFs and their weights are updated with Eq. (5). Moreover, it is very important in semi-supervised clustering to make full use of the information embedded in the labeled objects. The details of the k-means based clustering algorithms are as follows:

#### Feature-space-seed k-means (FSSK-means):

The labeled sample is used to initialize the k-means algorithm. Rather than initializing k-means randomly, the *k*th cluster is initialized with the mean of the kth partition of the labeled set. The labeled samples are only used for initialization, and they are not used in the following steps of the algorithm, such as re-estimating means and reassigning clusters in k-means clustering procedures. Taking into consideration on our specific application domain (text clustering), we utilized the classical similarity measurement cosine coefficient:

(8) $$S(X,Y)=|X\cap Y|∕({|X|}^{1∕2}{|Y|}^{1∕2})$$

where *X* and *Y* are two texts. *FSSK-means* is based on the semi-supervised k-means algorithms proposed by (Basu et al. 2002). *FSSK-means* inherits the labeled sample learning strategy, but adds the utilization of unlabeled samples with a strict feature space learning strategy (such as trust sample and *RIF* selection).

#### Feature-space-constrained k-means (FSCK-means):

The labeled sample set is also used to initialize the k-means algorithm as described for *FSCK-means*. However, in the subsequent procedures of *FSCK-means*, the labeled samples are employed in the clustering phase. This means that when the algorithm needs to re-estimate means and reassign clusters, the labeled samples join in the computation. In contrast, labeled samples in *FSSK-means* are only used in initialization. Equation (8) is also used in *FSCK-means* to measure the similarity.

### 2.2 Affinity propagation based FSL models

The core idea of affinity propagation (AP) is that it can be viewed as a method that searches for the minima of an energy function:

(9) $$G(x)=-\sum_{i=1}^{N}s(d_{i},c_{k})$$

where *N* is the data number, *k* is the cluster index number, *d_i_* is a sample involved in cluster *k* and *c_k_* is the cluster center.

After the AP clustering processes, we consider the sample as a trust sample according to the above AP’s objective function and Eq. (1):

(10) $$d^{*}=\underset{1\leq i,k\leq N}{\arg\;\min}[-(s(i,k)+s(k,k))]$$

where *s*(*i*, *k*) is the similarity between sample *d_i_* and its center *c_k_* and *s*(*k*, *k*) is the priori suitability of point *c_k_* to serve as an exemplar. Similarly, Eq. (2) is used to search for the RIFs and Eq. (5) is used to update their weights.

*Feature-space affinity propagation* (*FSAP*) is performed on the basis of AP clustering. Labeled samples are directly used during the responsibility and availability messages transmission. It utilizes the cosine coefficient to measure the similarity between documents, and the self-similarity utilizes:

(11) s(i,i)={P(x)−ϕ(Q(x)−P(x))+∞,N<i≤M}

where *N* is the scale of the unlabeled data set, *M* indicates the scale of all the labeled and unlabeled samples, $P(x)=\min_{1\leq i,j\leq N,i\neq j}\{s(i,j)\}$, $1<i\leq N,Q(x)=\max_{1\leq i,j\leq N,i\neq j}\{s(i,j)\}$, 1 < *i* ≤ *N* and *φ* is an adaptive factor. Moreover, in the FSL frame, we use Eq. (10) to select the trust samples. Then, with the rules of Eqs. (2) and (3), FSAP achieves the updated feature space.

*Feature-space-seeds affinity propagation* (*FSSAP*) *FSSAP* is derived from the combination of seeds affinity propagation (SAP) (Guan et al. 2011) and the FSL model. SAP related concepts are introduced: For document *d_i_*, we denote *F_i_* as the feature set of *d_i_*, and *SF_i_* as the significant feature set of *d_i_*, including the most significant features–such as the words and key-phrases in title and abstract of *d_i_*. Then for two document samples *d_i_* and *d_j_*, the co-feature set *CFS*_(*i*,*j*)_, unilateral feature set *UFS*_(*i*,*j*)_, and significant co-feature set *SCS*_(*i*,*j*)_ are defined as follows:

(12) $$CFS_{\left(i,j\right)}=\{f|f\in Fi\;and\;f\in F_{j}\}$$

(13) $$UFS_{\left(i,j\right)}=\{f|f\in Fi\;and\;f\notin F_{j}\}$$

(14) $$SCS_{\left(i,j\right)}=\{f|f\in Fi\;and\;f\in SF_{j}\}$$

FSSAP inherits the tri-set similarity measurement from SAP:

(15) $$s(i,j)=\alpha \sum_{m1}^{|CFS|}n_{j}^{m}+\beta \sum_{q=1}^{|SCS|}n_{SF_{j}}^{q}-\gamma \sum_{p=1}^{|UFS|}n_{i}^{q}$$

where ∣ ∙ ∣ indicates the scale of a set; $n_{j}^{m}$ and $n_{i}^{q}$ have a frequency of *f_q_* in *SF_j_*, and *α*, *β* and *γ* are adaptive factors that have been extensively discussed in Guan et al. (2011). The labeled samples are first merged into compact seeds based on the labeled information before putting labeled and unlabeled samples together. Then, the compact seeds are entered into the message-passing of AP. While in the learning process, it should be noticed that the features are divided into a normal feature set and a significant feature set. Denote *FC_k_* as the normal feature set of cluster k, and *SFC_k_* as the significant feature set of cluster *k*. Then after obtaining the trust sample d* of cluster *k*, if a feature of Eq. (2) is satisfied, it should be added into *FC_k_*. Moreover, if it satisfies

(16) $$m_{SF_{k}}^{i}\geq \sum_{p=1}^{SFC_{k}}m_{SF_{k}}^{p}∕|SFC_{k}|$$

then it should be added into *SFC_k_*, where $m_{SF_{k}}^{i}$ represents the frequency of feature *f_i_* in *SFC_k_*. Comparing FSAP and FSSAP, the most different strategy between them is that the latter chooses tri-set similarity measurement and compact seeds.

To compare the introduced ten clustering algorithms, Table 2 depicts all the different strategies. The main distinction among between semi-supervised learning and FSL laid on the different assumptions. The former only focus on the performance improvement. On the contrary, the latter not only consider the performance but also optimize the feature space. It is a bi-objective optimization.

## 3 Results and discussion

### 3.1 Datasets and evaluation

All the classical clustering, semi-supervised learning and new proposed FSL algorithms are applied to two benchmark text datasets: Reuters-21578 (Reuters) and 20 Newsgroups (20NG). They are maintained in the UC Irvine Machine Learning Repository and are widely used (Asuncion and Newman 2007; Bekkerman et al. 2003). Moreover, for text data clustering and classification, the high-dimensional and sparse matrix computation is a typical problem (Guan et al. 2011). FSL model transfers the original feature space into compact ones which can solve this problem.

The publicly available Reuters dataset is a widely used benchmark text mining data set which is pre-classified manually (Lewis 2004). This class information is eliminated before learning, and is used to evaluate the performance of each algorithm at the end. The original Reuters data consist of 22 files (for a total of 21,578 documents) and contains special tags like < *TITLE* >,< *TOPICS* >, and < *DATE* > among others, which are the text information and introduction. We firstly cut the files into a series of single texts and strips the documents from the special tags. Then, those documents which belong to at least one topic are selected. To avoid the imbalanced data problem in Reuters, the top 10 classes are selected as other researches (Estabrooks et al. 2004; Guan et al. 2011).

20NG is also a widely used benchmark data set. It is collected by Ken Lang and contains 19997 texts from 20 news groups (Rennie 2008). The original 20NG contains a large number of headers information (such as: Newsgroups, Subject, and Date) in each document. These headers are deleted before the experiments to avoid introducing label information.

The pre-processing includes text extraction, stop words removal and word frequency computation for each document, the data sets were changed into the superset form in Step 0 of Fig. 1. The labeled samples used in all of the algorithms are randomly selected without any prior knowledge. The count of the unlabeled data is 400. To pursue the compact feature space, for Reuters, the count of the labeled data is from 10 to 400 (2.5 to 50%); for 20NG, the count is from 20 to 400 (5 to 50%).

To evaluate the performance of clustering, two types of measures were applied, namely F-measure and entropy, which has been widely used in information retrieval. They are used to compare the generated result with the set of categories created by experts. The F-measure is a harmonic combination of the precision and recall values. The larger the F-measure is, the better is the clustering performance. Entropy provides a measure of the uniformity or purity of a cluster. In other words, it can tell us how homogeneous a cluster is. The smaller the Entropy is, the better the clustering performance.

### 3.2 FSL vs. semi-supervised algorithms

To examine the effectiveness of the proposed model, several existing algorithms were implemented for comparison, the blue line represents k-means algorithm, the green line is SK-means, the red line CK-means, the cyan line is FSSK-means and the purple line is FSCK-means. The x axes indicate the percentage of labeled samples in the datasets, the y axes of Fig. 2a, c are the maximum F-measure for each labeled sample scales, and the y axes of Fig. 2b, d are the minimum Entropy for each labeled sample scales. From Fig. 2, we can clearly see that the FSL learning curves (FSCK-means in purple line and FSSK-means in cyan line) are the highest F-measure and lowest entropy. They beat the other three algorithms on different labeled sample scales.

The experiments show that our feature space learning algorithms outperform the classical k-means and semi-supervised k-means. The effectiveness of the FSL model can be seen in the distance between the classical semi-supervised clustering and feature space learning curves. We compute the mean value for each algorithm’s learning curve in Fig. 2 and the numerical values of the maximum/minimum of these k-means based algorithms. They are showed in Table 3. From Table 3, we can see FSL based k-means algorithms are superior to the semi-supervised k-means clustering algorithms. FSCK-means achieved the highest F-measure (0.614 and 0.342 for Reuters and 20NG, respectively) and lowest Entropy (0.445 and 0.815 for Reuters and 20NG, respectively). For the mean value of each learning curve, it can be found that the FSL algorithms also achieved the highest mean F-measure (0.546 and 0.289 for Reuters and 20NG, respectively) and the lowest entropy (0.533 and 0.889 for Reuters and 20NG, respectively). All the two FSL algorithms are performed better than the ones without FSL strategies, e.g. FSSK-means gets 13.8% higher F-measure than SK-means and 73.6% higher than k-means (0.197) on 20NG data.

**Fig. 3:**
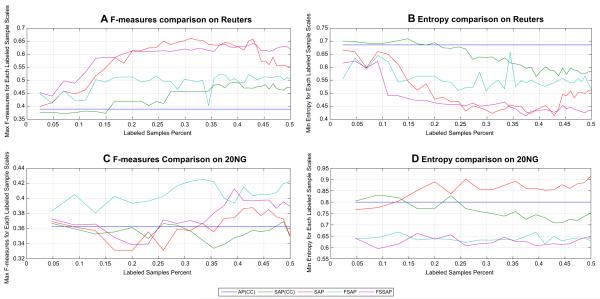
Comparison results of AP based methods

The comparison results of AP based algorithms are depicted in Fig. 3. In Fig. 3, the blue line represents AP(CC) algorithm, the green line is SAP(CC), the red line SAP, the cyan line is FSAP and the purple is FSSAP. The x axes indicate the percentage of labeled samples in the datasets, the y axes of Fig. 3a, c are the maximum F-measure for each labeled sample scales, and the y axes of Fig.3b, d are the minimum Entropy for each labeled sample scales. In Fig. 3a, b, because SAP (red line) used the tri-set similarity, its learning curve is near FSSAP (purple line) on Reuters. However, on 20NG the gap between FSSAP and SAP is enlarged in Fig. 3c, d. In addition, on 20NG, FSAP achieved the highest F-measures in Fig. 3c. In summary, the FSL models FSAP and FSSAP broadly performed better than those without. Combined with the results of k-means based algorithms, it illustrates that the FSL model is not sensitive to the exact algorithms and can be used in a variety of clustering methods.

From the above experiments and results, it can be seen that the FSL model based algorithms could achieve better results than the traditional semi-supervised clustering algorithms on different data sets. It is because that by updating the feature space, the FSL model could modify the clustering strategy and embed information in similarity measurement simultaneously. The superior experimental results show that FSL can find out those important features and construct an informative feature space. Moreover, the FSL model can combine with different clustering algorithms not limited to the k-means and affinity propagation.

### 3.3 FSL vs. incremental semi-supervised algorithm

The incremental affinity propagation (IAP) is an existing AP based semi-supervised algorithm (Shi et al. 2009) which can be considered as SAP (CC) plus an incremental trust sample selection process, while FSAP is SAP (CC) combined with FSL. To test the only effect of the feature space learning procedure, the result of FSAP is compared with that of IAP in Shi et al. 2009. The F-measure and entropy comparison results are shown in Tables 4 and 5.

Tables 4 and 5 show that FSSAP with feature space learning receives the best values (the maximum F-measures and minimum entropies) in most cases, i.e. FSSAP performs better than IAP on five data scales (10, 100, 200, 300, and 400) on both F-measure and entropy. Most importantly, FSSAP gets 14.3% higher F-measure than IAP with a data scale of 100 and a 13.5% lower entropy score than IAP at 200.

The main difference between FSAP and IAP is that the former adopts the FSL model, which employs the more stringent feature space learning strategy. On the contrary, IAP simply adds unlabeled samples into clustering without selection and feature space promotion. Although IAP can obtain similar or even better results at the beginning, because of the noise accumulation and semantic shift, it quickly falls behind FSAP and FSSAP.

### 3.4 Learned feature spaces

During the clustering procedures, the FSL model also spans new feature spaces and updates feature values. We picked out the feature spaces of FSCK-means on Reuter data to illustrate the transformed feature spaces.

From Table 6, it can be seen that the feature space has been changed after performing FSCK-means clustering. At the start-up, all the documents should be represented as a vector based on the whole vector space of the data. Because most of the documents do not contain all the words used in the data, the vector space model often falls into high-dimensional and sparse. For example, in our case it consists of 8277 dimensions. However, with the FSL model, we selected no more than 3500 features (the largest one is 3418 for Cluster 9) that could represent all these documents and clusters. The newly generated feature spaces are only 13.4–41.3% of the original one. Moreover, during the clustering, the values of the features are also accurately refined. For example, the “trade” value in cluster 9 in Table 6 is 4.97. This cluster coincides with the “trade” class in Reuters. With these well-refined feature spaces, more accurate classifiers can be constructed easily by similarity or distance computing.

### 3.5 Feature spaces visualization

**Fig. 4:**
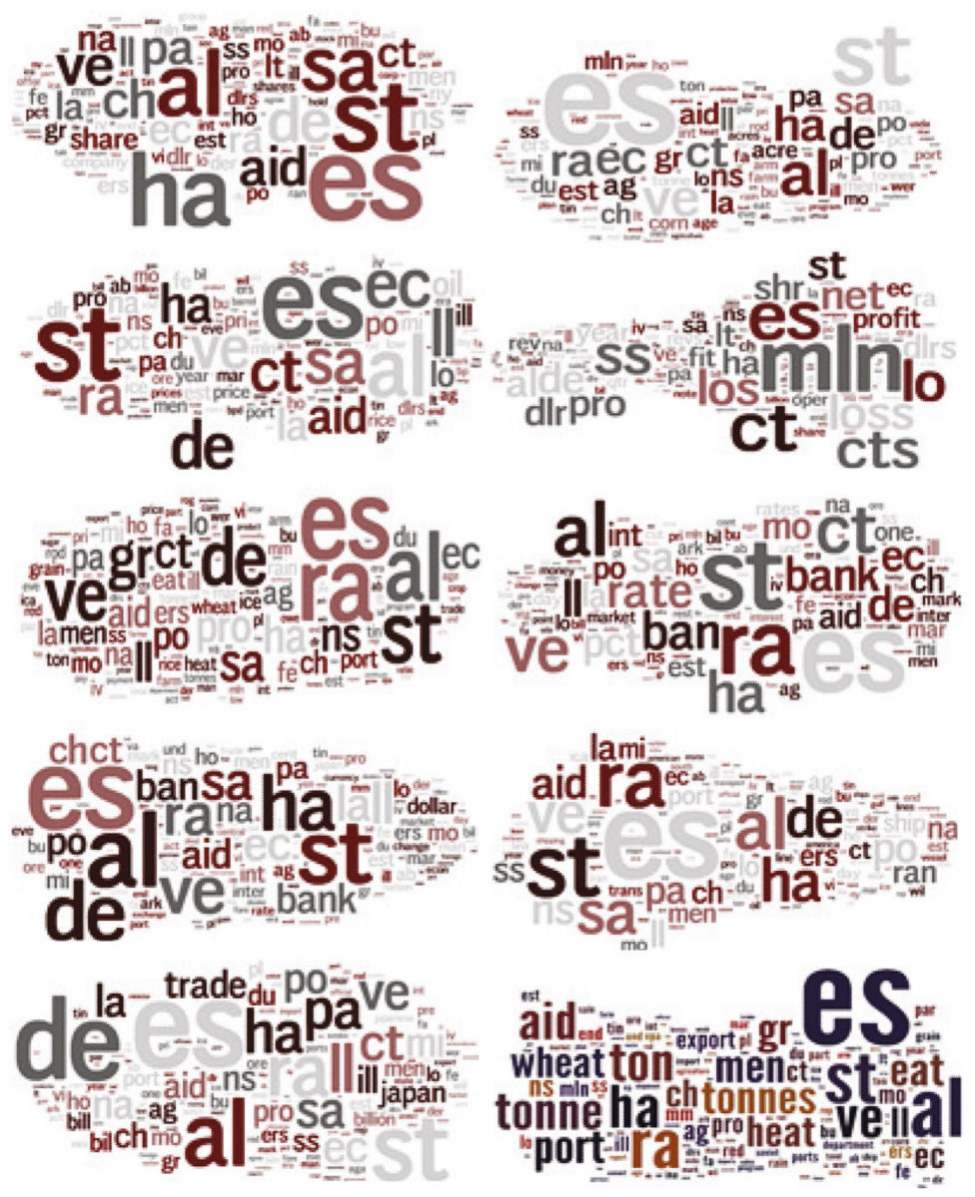
Visualization of feature space

Different from the abstract concepts of representation learnning, we make a visualization example for the learned feature space to make it visible and easy understand. In Fig. 4, the learned feature spaces of FSCK-means on Reuter data have been depicted. Instead of building up the whole vector spaces of the data and the high-dimensional and sparse matrix computing, with the FSL model, a much smaller feature space is constructed by those few labeled samples and the refinement of unlabeled samples features. At last, the learned feature spaces with low dimension could represent all the documents and clusters. Moreover, during the clustering, the values of the features are also more accurately depicted. With the well-refined feature spaces, the word clouds of the learned feature spaces for each cluster are drawn. The larger a feature value is, the bigger its logo. In addition, more accurate classifiers based on these learned feature spaces could be easily constructed by similarity or distance computing.

## 4 Conclusion

In this paper, we proposed a feature space learning model and four FSL algorithms. Inspired by Zipf’s law and words bursts, FSL model employs risk control strategies to avoid untrusted samples and filter the features. By constructing a more powerful feature space, the four clustering algorithms perform better than the classical clustering (MacQueen et al. 1967; Frey and Dueck 2007), semi-supervised clustering (Basu et al. 2002; Guan et al. 2011) and even incremental semi-supervised algorithms (Shi et al. 2009), e.g. on F-measure, FSCK-means is 73.6% higher than k-means and FSSAP gets 14.3% higher than IAP. Experimental results on the benchmark datasets demonstrate that the FSL model can dynamically promote learning performance and construct better understandable feature spaces. However, to pursue feature space and clusters simultaneously, the computation complexity of FSL model is higher than classical clustering and semi-supervised clustering models. Further model innovations may be needed to addressing this remaining limitation.

## Figures and Tables

**Table 1:** Pros and cons of the introduced models

Models	Pros and Cons	References
Deep learning algorithms	Pros: They can explore feature space and learning good representations. Cons: Deep architectures are too challenging to train effectively and lack of inter- pretability	Deep belief networks (Jiang et al. 2016), auto-encoders (Hinton and Salakhutdinov 2006), convolutional neural network (Esteva et al. 2017) and recurrent neural networks (Zhang et al. 2018; Karpathy et al. 2015)
Clustering	Pros: Only using the unlabeled sources can detect intrinsic distribution. Cons: Only using the unlabeled sources is lack of guidance for special aim	Recommendation systems (Bobadilla et al. 2013), public opinion analyses, (Semetko and Valkenburg 2000), and information retrieval areas (Frakes and Baeza-Yates 1992), image segmentation, object recognition, video tracking, and etc. (Jain et al. 1999; Huang et al. 2018; Wu et al. 2018; Guan et al. 2011)
Semi-supervised clustering	Pros: Use the information both from labeled data and unlabeled data. Cons: Unlabeled data should be explored carefully and it performed without feature learning procedure	Li and Zhou (2015); Tang et al. (2007); Wang et al. (2012); Guan et al. (2011); Shi et al. (2009); Xue et al. (2011)

**Table 2:** Different learning strategies for related algorithms

	Tri-Set similarity	Semi-supervised	FSL
k-means	×	×	×
AP(CC)	×	×	×
SK-means	×	✓	×
CK-means	×	✓	×
SAP (CC)	×	✓	×
SAP	✓	✓	×
FSSK-means	×	✓	✓
FSCK-means	×	✓	✓
FSAP	×	✓	✓
FSSAP	✓	✓	✓

**Table 3:** Semi-supervised K-means VS FSL K-means

Data	Evaluation	SK-means	CK-means	FSSK-means	FSCK-means
Reuters	Max-F	0.517	0.600	0.542	**0.614**
	Min-E	0.563	0.494	0.547	**0.445**
	Mean-F	0.462	0.509	0.477	**0.546**
	Mean-E	0.619	0.576	0.611	**0.533**
20 Newsgroup	Max-F	0.289	0.336	0.329	**0.342**
	Min-E	0.870	0.825	0.841	**0.815**
	Mean-F	0.250	0.288	0.276	**0.289**
	Mean-E	0.936	0.890	0.906	**0.889**

The best results are in bold

**Table 4:** FSL AP vs IAP on F-measure

	10	50	100	200	300	400
IAP	0.456	0.465	0.449	0.459	0.465	0.465
FSAP	**0.503**	0.423	0.513	0.500	0.514	0.503
FSSAP	0.452	**0.532**	**0.612**	**0.621**	**0.642**	**0.618**

The best results are in bold

**Table 5:** FSL AP vs IAP on entropy

	10	50	100	200	300	400
IAP	0.698	0.594	0.594	0.598	0.598	0.596
FSAP	**0.555**	0.621	0.565	0.517	0.540	0.512
FSSAP	0.616	**0.490**	**0.463**	**0.466**	**0.427**	**0.433**

The best results are in bold

**Table 6:** Learned feature spaces

Algorithms	Clusters	Features’ count	Example features
FSCK-means	1	2434	Aid = 3.37; share = 1.84; men = 1.72; ers = 1.54; dlr = 1.52
	2	2233	Pro = 3.93; pro = 3.93; aid = 3.71; mln = 3.07; est = 3.53; acre = 2.89
	3	3364	Aid = 3.37; aid = 6.02; ill = 3.52; pct = 3.11; pro = 3.08; pri = 2.70
	4	1109	Aid = 3.37; mln = 3.64; loss = 1.93 net = 1.70; pro = 1.55; dlr = 1.51
	5	2633	Aid = 3.37; pro = 3.66; aid = 3.13; ers = 2.49; men = 2.44; eat = 2.17
	6	2281	Aid = 3.37; ban = 3.79; rate = 3.66; bank = 3.47; pct = 3.11;aid = 2.56
	7	2652	Aid = 3.37; ban = 4.56; aid = 4.47; bank = 4.42; int = 2.65;dollar = 2.45
	8	2956	Aid = 3.37; aid = 3.66; ran = 2.67; ers = 2.47; men = 2.27; port = 2.19
	9	**3418**	Trade = 4.97; ill = 4.79; aid = 4.67; pro = 4.05; Japan = 3.85;
	10	2317	Ton = 3.40; aid = 3.23; tonnes = 2.88; eat = 2.85; wheat = 2.63;
